# Isolation and Characterization of Human Monoclonal Antibodies to Pneumococcal Capsular Polysaccharide 3

**DOI:** 10.1128/Spectrum.01446-21

**Published:** 2021-11-10

**Authors:** Rachelle Babb, Christopher R. Doyle, Liise-anne Pirofski

**Affiliations:** a Division of Infectious Diseases, Department of Medicine, Albert Einstein College of Medicine & Montefiore Medical Center, Bronx, New York, USA; b WCG IBC Services, Puyallup, Washington, USA; Emory University School of Medicine

**Keywords:** *Streptococcus pneumoniae*, agglutination, immunology, immunotherapy, monoclonal antibodies

## Abstract

The current pneumococcal capsular polysaccharide (PPS) conjugate vaccine (PCV13) is less effective against Streptococcus pneumoniae serotype 3 (ST3), which remains a major cause of pneumococcal disease and mortality. Therefore, dissecting structure-function relationships of human ST3 pneumococcal capsular polysaccharide (PPS3) antibodies may reveal characteristics of protective antibodies. Using flow cytometry, we isolated PPS3-binding memory B cells from pneumococcal vaccine recipients and generated seven PPS3-specific human monoclonal antibodies (humAbs). Five humAbs displayed ST3 opsonophagocytic activity, four induced ST3 agglutination *in vitro*, and four mediated both activities. Two humAbs, namely, C10 and C27, that used the same variable heavy (V_H_) and light (V_L_) chain domains (V_H_3-9*01/V_L_2-14*03) both altered ST3 gene expression *in vitro*; however, C10 had fewer V_L_ somatic mutations, higher PPS3 affinity, and promoted *in vitro* ST3 opsonophagocytic and agglutinating activity, whereas C27 did not. In C57BL/6 mice, both humAbs reduced nasopharyngeal colonization with ST3 A66 and a clinical strain, B2, and prolonged survival following lethal A66 intraperitoneal infection, but only C10 protected against lethal intranasal infection with the clinical strain. After performing V_L_ swaps, C10V_H_/C27V_L_ exhibited reduced ST3 binding and agglutination, but C27V_H_/C10V_L_ binding was unchanged. However, both humAbs lost the ability to reduce colonization *in vivo* when their light chains were replaced. Our findings associate the ability of PPS3-specific humAbs to reduce colonization with ST3 agglutination and opsonophagocytic activity, and reveal an unexpected role for the V_L_ in their functional activity *in vitro* and *in vivo*. These findings also provide insights that may inform antibody-based therapy and identification of surrogates of vaccine efficacy against ST3.

**IMPORTANCE** Despite the global success of vaccination with pneumococcal conjugate vaccines, serotype 3 (ST3) pneumococcus remains a leading cause of morbidity and mortality. In comparison to other vaccine-included serotypes, the ST3 pneumococcal capsular polysaccharide (PPS3) induces a weaker opsonophagocytic response, which is considered a correlate of vaccine efficacy. Previous studies of mouse PPS3 monoclonal antibodies identified ST3 agglutination as a correlate of reduced ST3 nasopharyngeal colonization in mice; however, neither the agglutinating ability of human vaccine-elicited PPS3 antibodies nor their ability to prevent experimental murine nasopharyngeal colonization has been studied. We generated and analyzed the functional and *in vivo* efficacy of human vaccine-elicited PPS3 monoclonal antibodies and found that ST3 agglutination associated with antibody affinity, protection *in vivo*, and limited somatic mutations in the light chain variable region. These findings provide new insights that may inform the development of antibody-based therapies and next-generation vaccines for ST3.

## INTRODUCTION

The current pneumococcal capsular polysaccharide conjugate vaccine PCV13 is less effective against Streptococcus pneumoniae serotype 3 (ST3) than against other vaccine-included STs. As a result, ST3 is a major cause of pneumonia and mortality in adults and children (1–5). Ample clinical data show that pneumococcal conjugate vaccination prevents pneumococcal colonization and transmission, with vaccine-elicited ST-specific opsonophagocytic serum antibodies generally considered a surrogate for vaccine efficacy (6–8). However, a relationship between vaccine-elicited opsonophagocytic antibodies and protection against ST3 has not been established. In addition, compared with other vaccine-included STs, the capsular polysaccharide of ST3 (PPS3) is poorly immunogenic and induces a weaker opsonophagocytic antibody response (2). This reduced immunogenicity has been attributed to the thick ST3 capsule (9) as well as the limited ability of PPS3 antibodies to clear ST3 via opsonophagocytosis *in vivo* due to large amounts of ST3 capsule shedding (10). Nevertheless, human and mouse opsonophagocytic PPS3 monoclonal antibodies (mAbs) that are protective in ST3 sepsis and pneumonia models in mice have been generated (11–15). Notably, an opsonophagocytic mouse mAb that protected against ST3 sepsis and pneumonia did not reduce ST3 colonization, whereas a nonopsonic mAb agglutinated ST3, reduced colonization, protected against sepsis and pneumonia, and altered ST3 gene expression *in vitro* and *in vivo* (11, 13, 16).

Bacterial agglutination, including that of the pneumococcus, is a long-recognized correlate of PPS antibody efficacy in experimental models (17, 18). While mouse and human PPS3 mAbs elicited by an experimental PPS3-tetanus toxoid (PPS3-TT) conjugate revealed that ST3 opsonophagocytosis and agglutination were mutually exclusive functions (11, 13, 16, 19), serum-derived antibodies to ST4 and ST23 exhibited both opsonophagocytic and agglutinating functions (20). Consistent with the latter finding, among a set of five PPS3 mouse mAbs generated in response to a PPS3-keyhole limpet hemocyanin (PPS3-KLH) conjugate, four exhibited both opsonophagocytic and agglutinating activity and only one mediated opsonophagocytosis (21). These findings suggest that the nature of PPS3 antibodies that mediate opsonophagocytosis and agglutination versus those that mediate one function and not the other may differ.

Reduced efficacy of PPS3-specific antibodies against ST3 disease has been attributed to impaired opsonophagocytic clearance, and it has been estimated that approximately 8 times more antibody is required to confer protection against ST3 based on the calculated correlate of protection for other pneumococcal STs (2, 10). Thus, deciphering the structural and functional characteristics of human vaccine-elicited PPS3 antibodies may advance our understanding of vaccine failure and facilitate development of antibody-based therapies and next-generation vaccines. To gain insight into the nature of human PPS3-binding antibodies, we generated PPS3 human mAbs (humAbs) from human pneumococcal vaccine recipients and determined their molecular derivation, PPS3 binding, and function *in vitro* and *in vivo*.

## RESULTS

### PPS3 humAbs use gene segments from the VH3 family.

**FIG 1:**
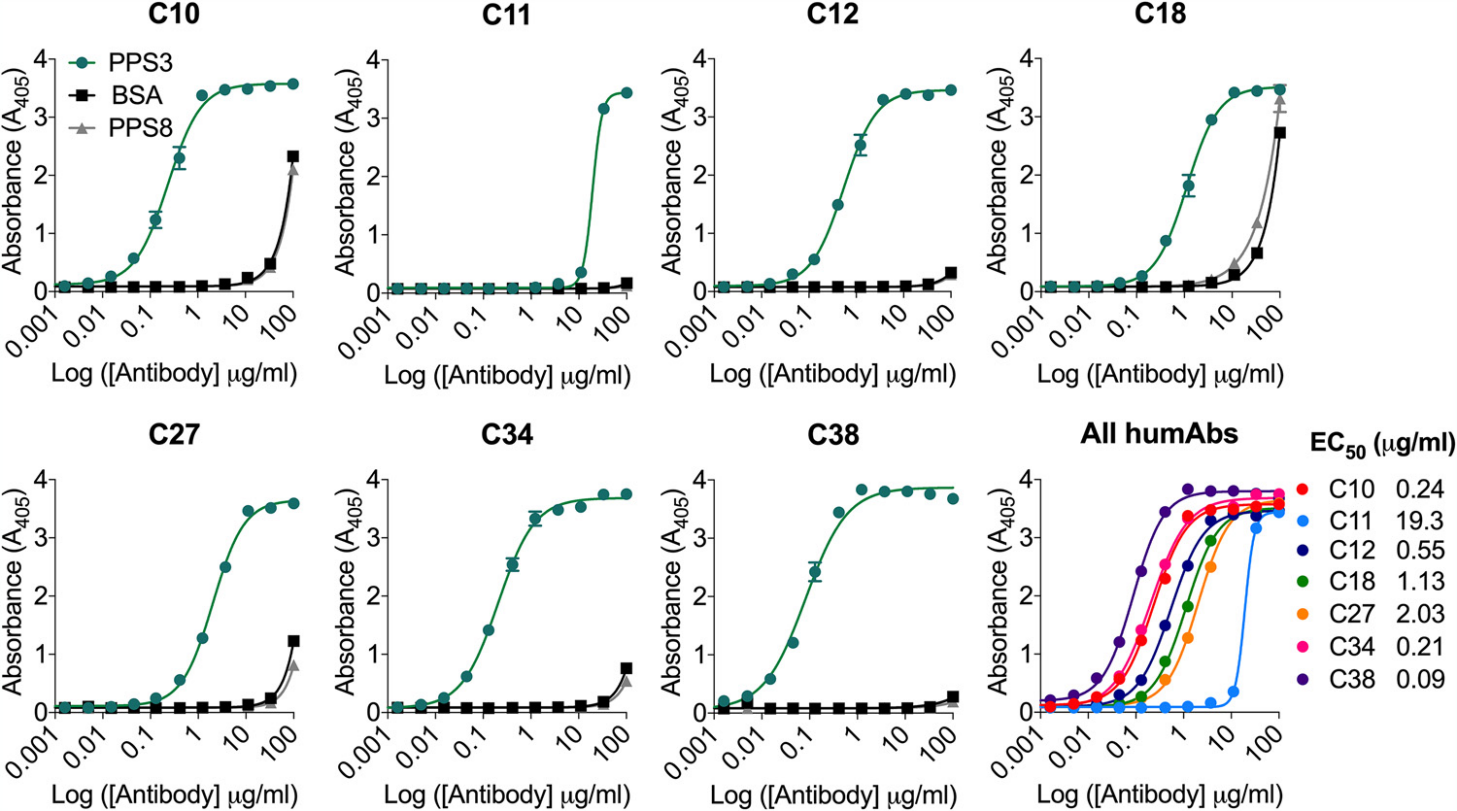
HumAb binding to pneumococcal polysaccharide 3 (PPS3) by ELISA. Binding as reflected by absorbance at 405 is shown on the *y* axis for the humAb concentrations shown on the *x* axis for each humAb. Results are representative of 3 independent experiments (*n* = 2). The numerical 50% effective concentration (EC_50_) for each humAb is indicated to the right of the panel depicting binding curves of all humAbs.

Seven PPS3-binding humAbs (PPS3 humAbs) were generated and tested for PPS3 binding by enzyme-linked immunosorbent assay (ELISA) (Fig. 1). C38 had the strongest binding to PPS3 (50% effective concentration [EC_50_], 0.09 μg/ml) followed by C34 (EC_50_, 0.21 μg/ml) and C10 (EC_50_, 0.24 μg/ml). Binding to a ST3 clinical strain, B2, was also similar by whole-cell ELISA and immunofluorescence (see Fig. S1 and S2 in the supplemental material). In addition, the humAbs did not bind to the cell wall polysaccharide (data not shown).

**FIG 2:**
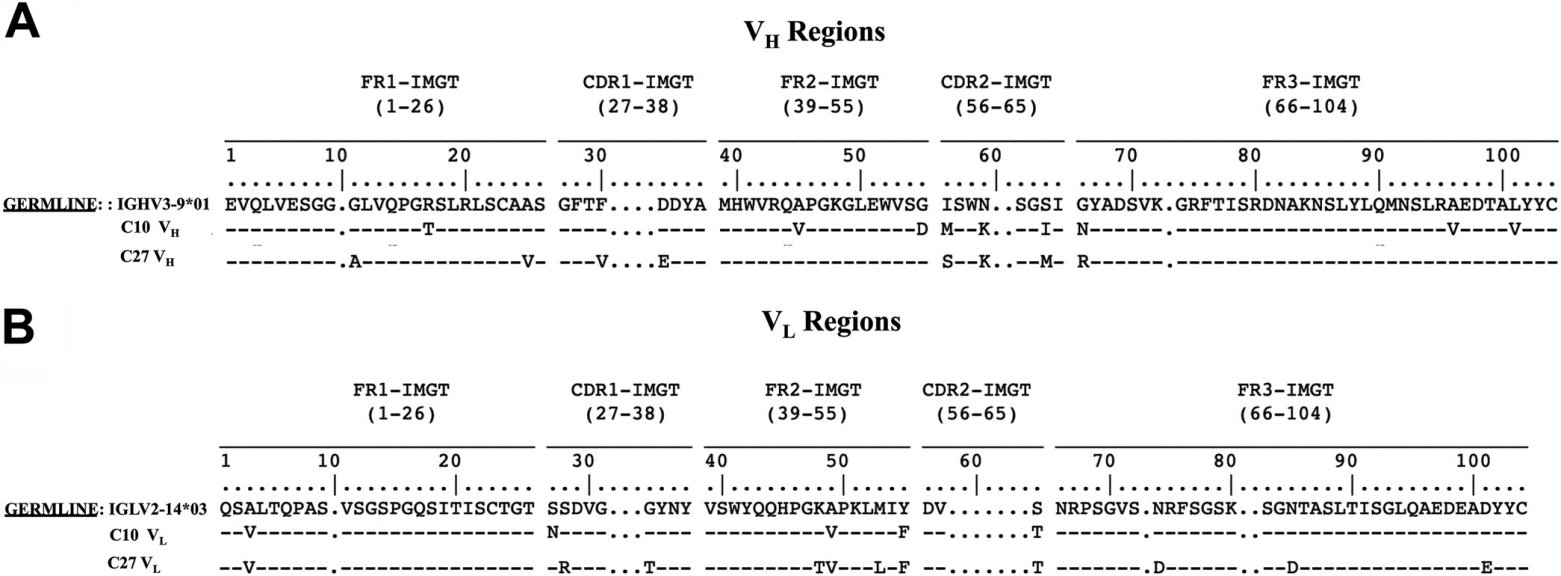
C10 and C27 variable heavy (V_H_) and variable light (V_L_) chain amino acid sequences. HumAb V_H_ (A) and V_L_ (B) sequences aligned with their germline counterparts based on IMGT/V-QUEST (sequence alignment software). Amino acid changes resulting from somatic mutations are indicated within the sequence alignment.

Sequencing analysis revealed that five humAbs (C10, C12, C27, C34, and C38) used lambda light chains (LCs) and two (C11 and C18) used kappa LCs. Based on IgBlast, six humAbs used variable heavy 3 (V_H_3) genes and one (C38) used a V_H_1 gene (Table 1). All seven humAbs had variable heavy (V_H_) and variable light (V_L_) complementarity-determining region (CDR) as well as framework region (FR) somatic mutations (see Fig. S3 and S4 in the supplemental material). In addition, all seven humAb CDR3s differed by sequence and length, but four (C10, C27, C38, and C11) had an Ala-Arg-Asp: ARD or Ala-Arg-Gly: ARG motif at the beginning of the V_H_ CDR3 region (Table 1). Two lambda humAbs, namely, C10 and C27, used the same heavy variable (V), diversity (D) and joining (J) (VDJ) gene segments and LC variable (V), joining (J) (VJ) gene segments, but their FRs and CDRs differed by several somatic mutations (Fig. 2). C10 and C27 had 9 and 8 V_H_ mutations, respectively, conferring amino acid changes relative to germline IGHV3-9*01, including four at the same positions and a shared lysine (K) in CDR2. C10 V_L_ was closer to germline IGVL2-14*03, with fewer mutations (5 versus 11) than C27, of which 4 were shared.

**TABLE 1:** Heavy and light chain VDJ gene usage and CDR3 sequences for all PPS3 humAbs

HumAb	LC	Heavy chain				Light chain		
		V gene	D gene	J gene	CDR3	V gene	J gene	CDR3
C10	λ	IGHV3-9*01	IGHD6-19*01	IGHJ6*04	A R D I E H A V N H P R M M V V	IGLV2-14*03	IGLJ2*01,IGLJ3*01	S S Y T R T N T L V
C27	λ	IGHV3-9*01	IGHD6-19*01	IGHJ6*04	A R D V A H A V N H P R I M S V	IGLV2-14*03	IGLJ2*01,IGLJ3*01,IGLJ3*02	T S Y T T D N T V I
C12	λ	IGHV3-23*04	IGHD6-19*01, IGHD7-27*01	IGHJ4*02	A K R P G D S T G W A F Y F E Y	IGLV4-69*01	IGLJ3*02	Q T W G T G R W V
C34	λ	IGHV3-72*01	IGHD2-8*02, IGHD3-9*01, IGHD6-13*01	IGHJ5*02	A R A T A W S F D P	IGLV2-14*01	IGLJ1*01	S S Y T S T Y I Y V
C38	λ	IGHV1-18*01	IGHD6-13*01	IGHJ4*02	A R G G I T T T G F D Y	IGLV1-51*02	IGLJ3*02	G A W D S S L N A G V
C11	κ	IGHV3-30*03	IGHD3-16*01, IGHD3-16*02	IGHJ4*02	A R G G K G L S G G D Y	IGKV2-28*01	IGKJ1*01	M Q A L Q T P W T
C18	κ	IGHV3-7*01	N/A	IGHJ4*02	G I G R L F Y	IGKV2-30*01	IGKJ2*01	M Q G T H W P Y T

### Some PPS3 humAbs bind PPS3 determinants recognized by mouse PPS3 mAbs.

To determine if the humAbs bind to similar or distinct PPS3 determinants, we performed a competition experiment with a PPS3 mouse IgG1 kappa mAb (1E2) (11, 13, 16). The results demonstrated that C10, C34, and C38 each compete with the mAb 1E2 for PPS3 binding, whereas C11, C12, C18, and C27 do not (see Fig. S5A in the supplemental material). Since C18 is a kappa antibody, whereas C10 and C27 are both lambda antibodies, we also performed another competition ELISA with these antibodies. The results showed that C10 competes with C18 for PPS3 binding, but C27 does not (see Fig. S5B in the supplemental material). This result suggests that C10 and C18 may recognize the same PPS3 determinant, whereas C27 does not.

### PPS3 humAbs agglutinate ST3 *in vitro*.

**FIG 3:**
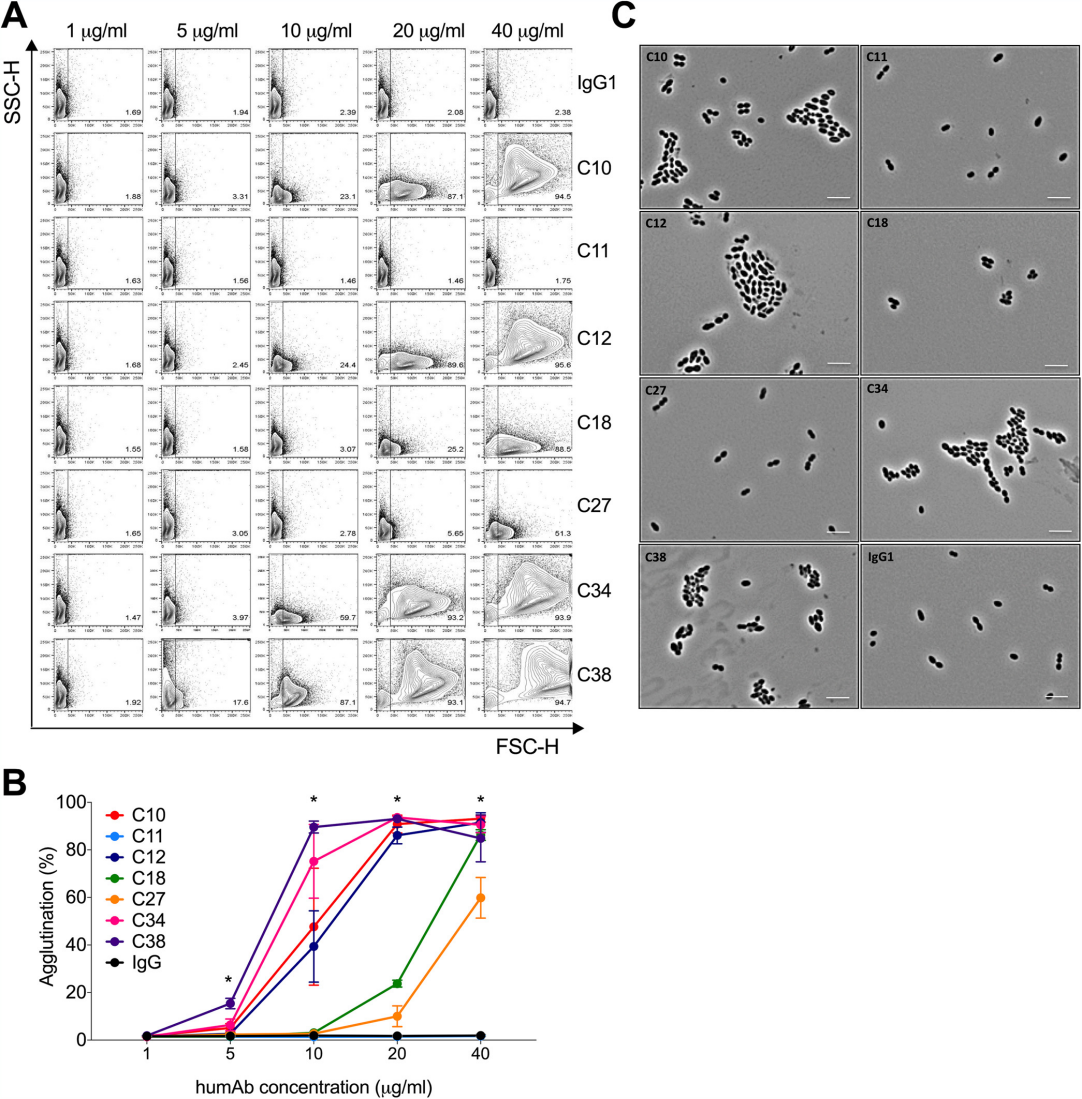
HumAb agglutination of ST3 A66. The ability of the humAbs to agglutinate ST3 (A66) was assessed by flow cytometry. (A) Representative fluorescence-activated cell sorter (FACS) dot plots showing the percent agglutination of all humAbs and control human IgG1 at various concentrations by flow cytometry. (B) Percentage of agglutination is shown on the *y* axis for different humAb concentrations indicated on the *x* axis. The graph represents data from 2 independent experiments (*n* = 2 per condition). (C) Light microscopy images of humAbs (20 μg/ml) with ST3 A66. Images at ×100 magnification are representative of 3 independent experiments (*n* = 2). Scale bars, 5 μm. Differences were determined by one-way ANOVA; at 5 μg/ml (C38 versus IgG1; ***, *P < *0.001), at 10 μg/ml (C34 and C38 versus IgG1; *, *P < *0.05), at 20 μg/ml (C10, C12, C18, C34, and C38 versus IgG1; **, *P < *0.01), and at 40 μg/ml (C10, C12, C18, C27, and C38 versus IgG1; ***, *P < *0.001).

**FIG 4:**
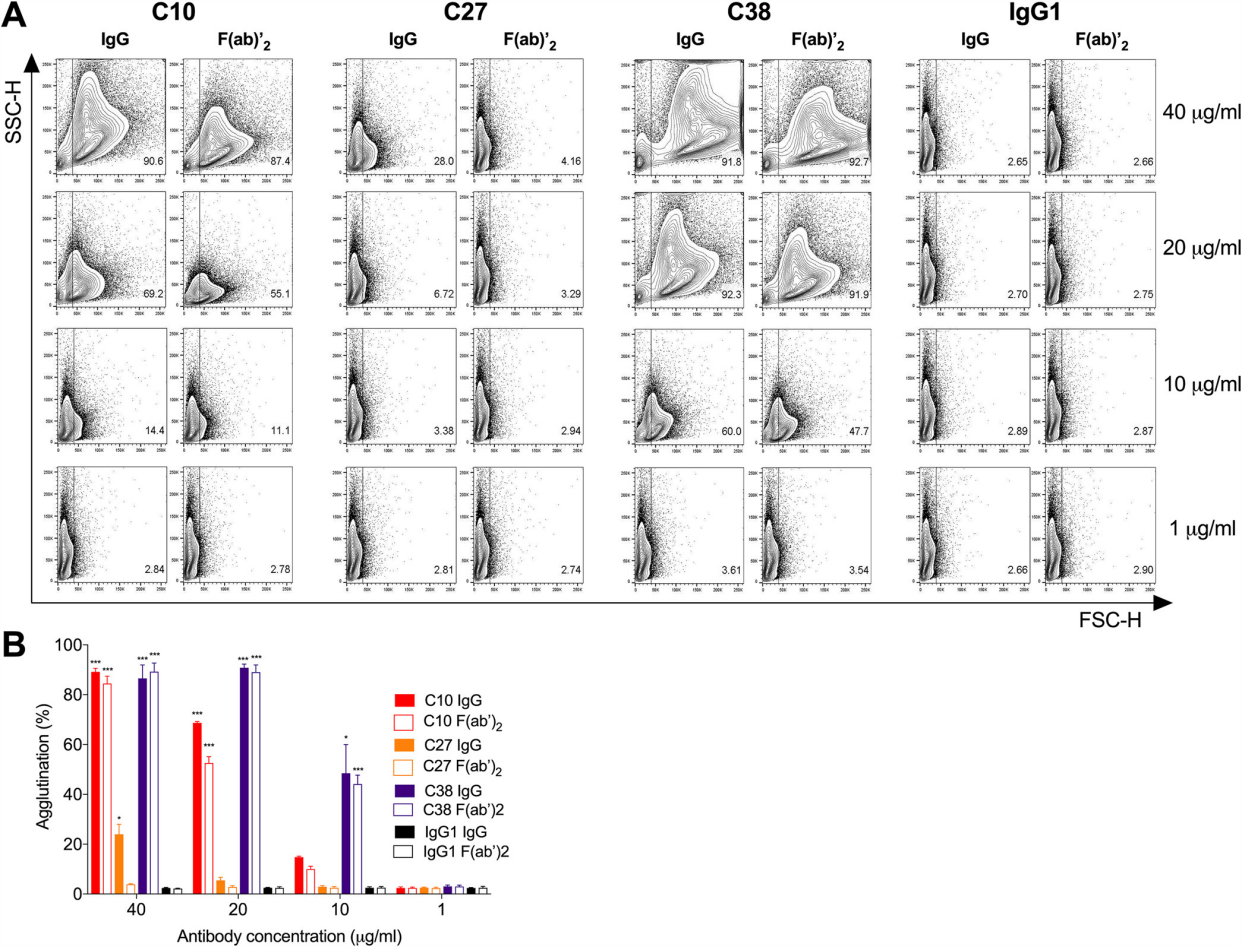
HumAb F(ab′)_2_ fragment agglutination of ST3 A66. The ability of whole IgG or F(ab′)_2_ fragments of humAbs (C10, C27, and C38) to agglutinate ST3 (A66) was assessed by flow cytometry. (A) Representative FACS dot plots showing the percent agglutination of the indicated whole humAbs, F(ab′)_2_ fragments, or control IgG1 at various concentrations. (B) Bar graph depicting percent agglutination on the *y* axis for whole humAb or F(ab′)_2_ fragment concentrations on the *x* axis. Results are representative of 2 independent experiments (*n* = 2 per condition). Differences were determined by one-way ANOVA; at 10 μg/ml (C38 IgG and C38 F([ab′]_2_ versus their respective IgG1 controls; *, *P < *0.05), at 20 μg/ml (C10 IgG, C10 F[ab′]_2_, C38 IgG, and C38 F[ab′]_2_ versus their respective IgG1 controls; ***, *P < *0.001), at 40 μg/ml (C10 IgG, C10 F[ab′]_2_, C38 IgG, and C38 F[ab′]_2_ versus their respective IgG1 controls; ***, *P < *0.001).

It has been reported previously that antibodies that agglutinate pneumococcus can reduce pneumococcal colonization (13, 22, 23). Thus, we determined the ability of the PPS3 humAbs to agglutinate ST3 A66 and the clinical strain B2 by flow cytometry and validated our findings with light microscopy. C10, C12, C34, and C38 each exhibited dose-dependent agglutination of ST3. At 10 μg/ml, C34 and C38 agglutinated ∼75% and 89% of bacteria, respectively, while C10 and C12 agglutinated ∼48% and 39%, respectively (Fig. 3A and B). Visual ST3 clumping was also observed with C10, C12, C34, and C38 by light microscopy (Fig. 3C). Similar results were obtained in agglutination experiments with the clinical strain B2 (see Fig. S6 in the supplemental material). F(ab′)_2_ fragments of C10 and C38 also agglutinated ST3 with levels comparable to their respective whole IgG (Fig. 4A and B).

### Opsonophagocytosis of ST3 by PPS3 humAbs.

**FIG 5:**
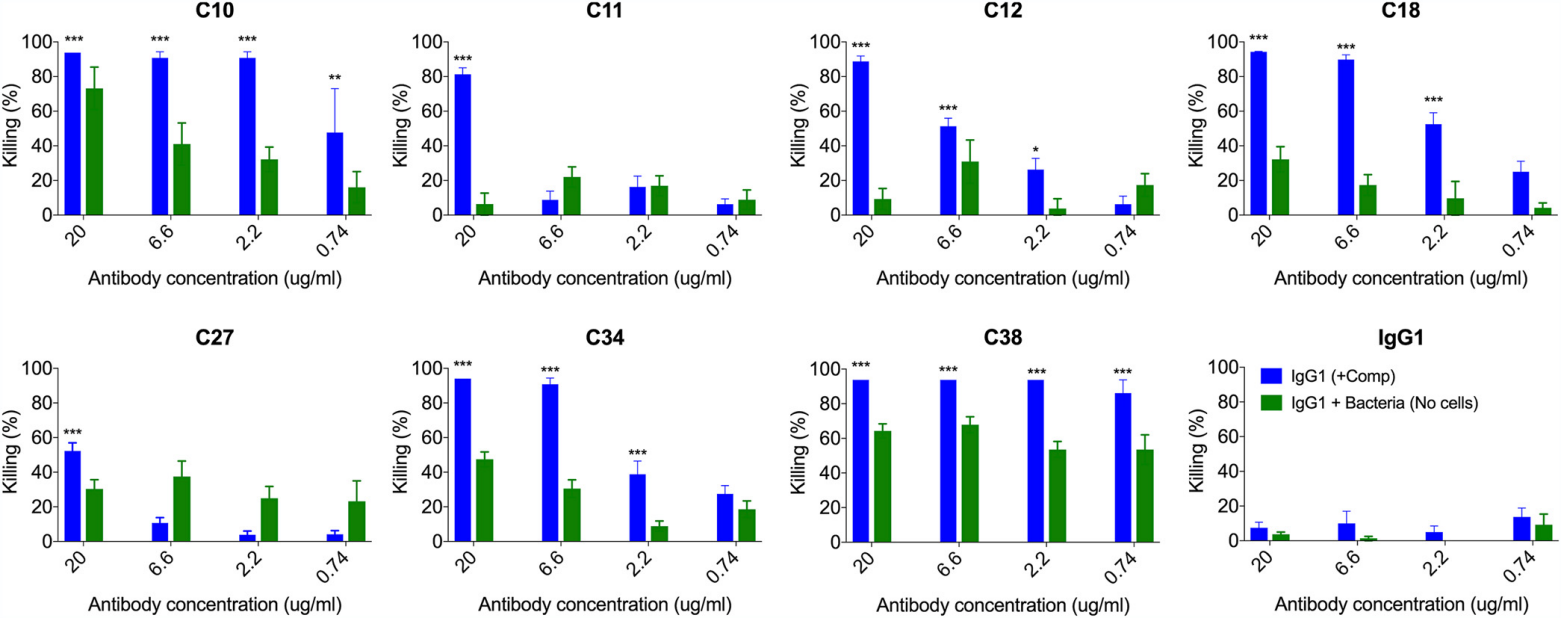
HumAb opsonophagocytic killing of ST3. HumAbs were tested for their opsonophagocytic killing activity with ST3 (A66) and HL60 cells. Percent killing is shown on the *y* axis for the different humAb concentrations shown on the *x* axis. Results are representative of 2 independent experiments (*n* = 4 per condition). *, *P < *0.05; **, *P < *0.01; ***, *P < *0.001 (one-way ANOVA) for humAbs versus IgG1 control.

The functional activity of the humAbs was determined with the standard opsonophagocytic assay (OPA) used in the field (24, 25). C10 and C38 displayed the highest activity with significant reductions in CFU at 0.74 μg/ml (Fig. 5) relative to the IgG1 control. C12, C18, and C34 reduced CFUs at 2.2 μg/ml and C11 and C27 at 20 μg/ml. When humAbs were incubated with ST3 without HL60 cells, C10, C18, C27, C34, and C38 reduced CFUs relative to the control. These reductions correlated with agglutination, except for that of C27.

### PPS3 humAbs reduce A66 and B2 nasopharyngeal colonization in C57BL/6 mice.

**FIG 6:**
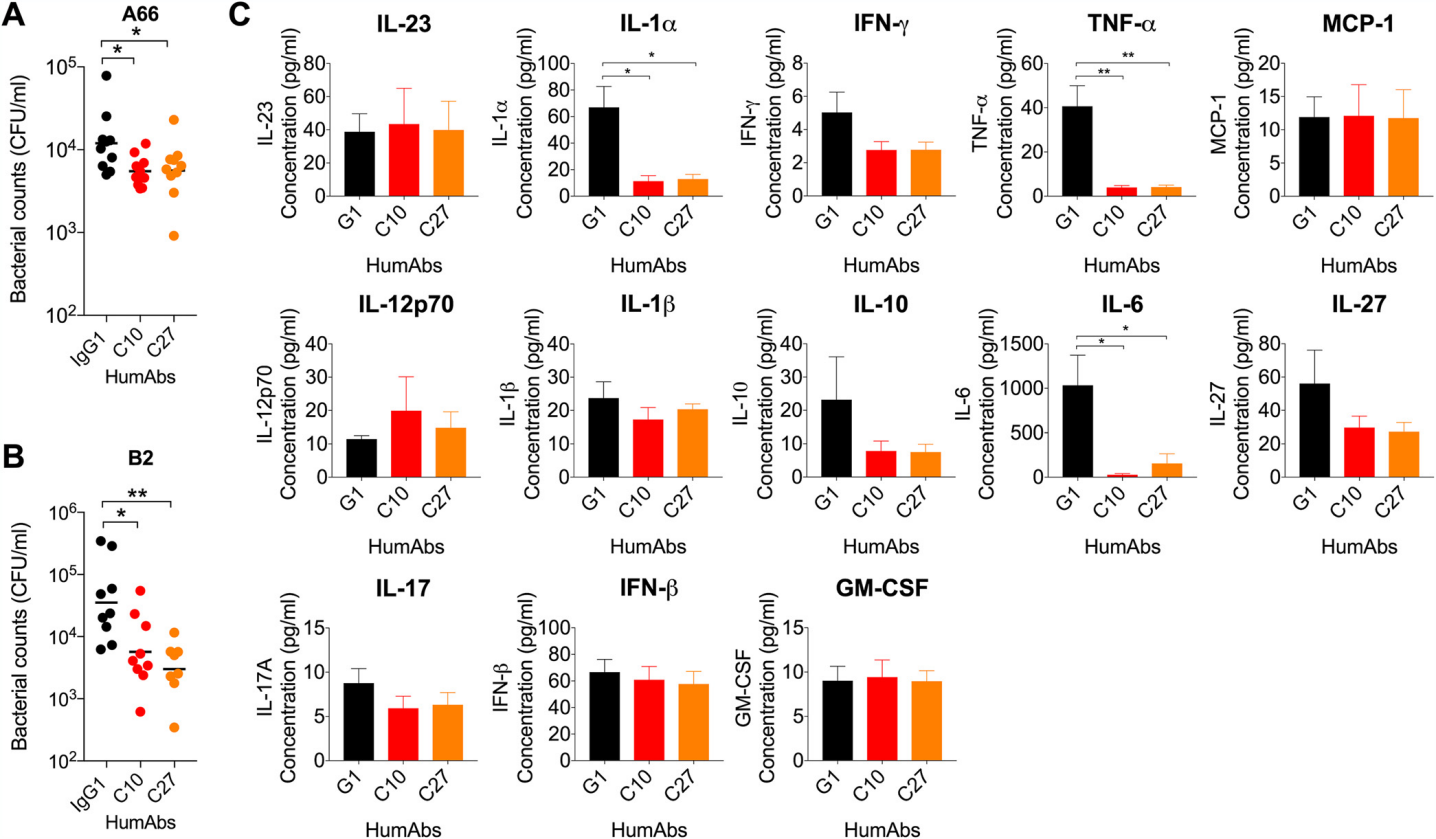
HumAb efficacy against ST3 colonization in C57BL/6 mice. HumAbs or a control IgG1 were administered i.n. in C57BL/6 mice 2 h before i.n. infection with 5 × 10^5^ CFU A66 (A) or 1 × 10^7^ CFU B2 (B). The nasal lavage CFU was enumerated 24 hours (A) or 4 days (B) postinfection. CFUs are depicted on the *y* axis for humAbs shown on the *x* axis. (C) Indicated cytokine concentrations via legendplex 4 days after infection of C57BL/6 mice with 1 × 10^7^ CFU B2 (B) are shown on the *y* axis for the humAbs on the *x* axis. Results are representative of 2 independent experiments (*n* ≥ 5 mice/group). *, *P < *0.05; **, *P < *0.01 (one-way ANOVA).

We next performed nasopharyngeal (NP) colonization experiments in mice with C10 and C27. These humAbs were chosen because they use the same V_H_3-9*01/V_L_2-14*03 gene elements but have different affinities and functional activities *in vitro*. Compared with the IgG1 control, administration of C10 and C27 reduced NP CFUs after infection with A66 (C10, *P* = 0.0388; C27, *P* = 0.0437) (Fig. 6A) and B2 (C10, *P* = 0.0128; C27, *P* = 0.0015) (Fig. 6B). CFUs were not detected in the lungs (data not shown). Compared with IgG1-treated controls, B2-infected C10- and C27-treated mice had significantly lower tumor necrosis factor alpha (TNF-α), interleukin-1α (IL-1α), and IL-6 levels in the NL at 4 days postinfection (Fig. 6C).

### PPS3 humAbs prolong the survival of mice lethally infected with A66 and B2.

**FIG 7:**
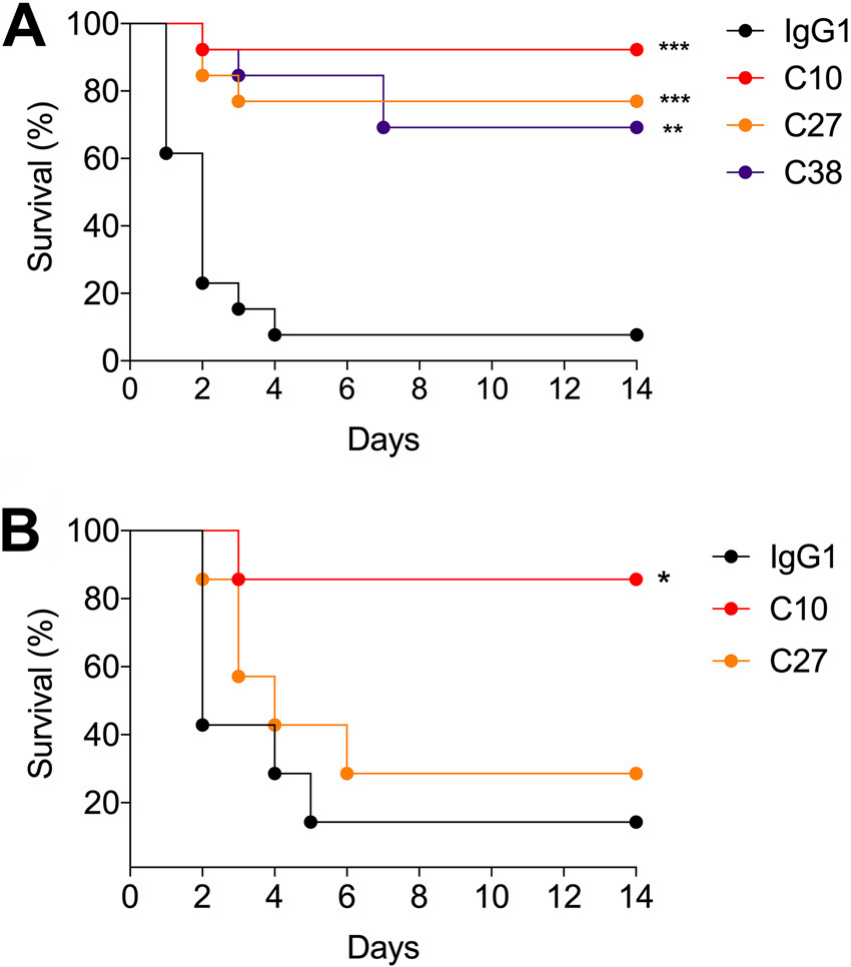
HumAb efficacy against lethal challenge with ST3 strains in C57BL/6 mice. (A) HumAbs or a control IgG1 were administered i.p. in C57BL/6 mice 2 h before i.p. infection with 5 × 10^5^ CFU A66 and then monitored for survival. (B) HumAbs or a control IgG1 were administered i.n. in C57BL/6 mice 2 h before i.n. infection with 5 × 10^7^ CFU B2 and monitored for survival. All curves show percent survival on the *y* axis for the indicated HumAbs monitored over 14 days shown on the *x* axis. Results are representative of 2 independent experiments (*n* ≥ 7 mice/group). *, *P < *0.05; **, *P < *0.01; ***, *P < *0.001, (Fisher's exact test).

The efficacy of C10, C27, and C38 was next investigated in lethal ST3 infection models. C38 was included because it exhibited strong ST3 binding, opsonophagocytosis, and agglutination. First, we analyzed the efficacy of our humAbs with A66. Intraperitoneal (i.p.) administration of all three humAbs prolonged survival after i.p. infection with A66 (Fig. 7A). C10 was the most protective (92% survival; *P* = 0.0001) followed by C27 (76%; *P* = 0.001) and C38 (70%; *P* = 0.0036). We next investigated humAb efficacy in a lethal intranasal (i.n.) infection model. Given that the lethal dose for A66 was 1 × 10^8^ CFU and that of the clinical strain B2 was 5 × 10^7^ CFU, we investigated the efficacy of humAbs with B2. Administration (i.n. route) of C10 but not C27 prolonged survival after lethal i.n. infection with B2 (85%; *P* = 0.0291) compared with the IgG1 control (Fig. 7B).

### PPS3 humAbs alter bacterial gene expression *in vitro*.

**FIG 8:**
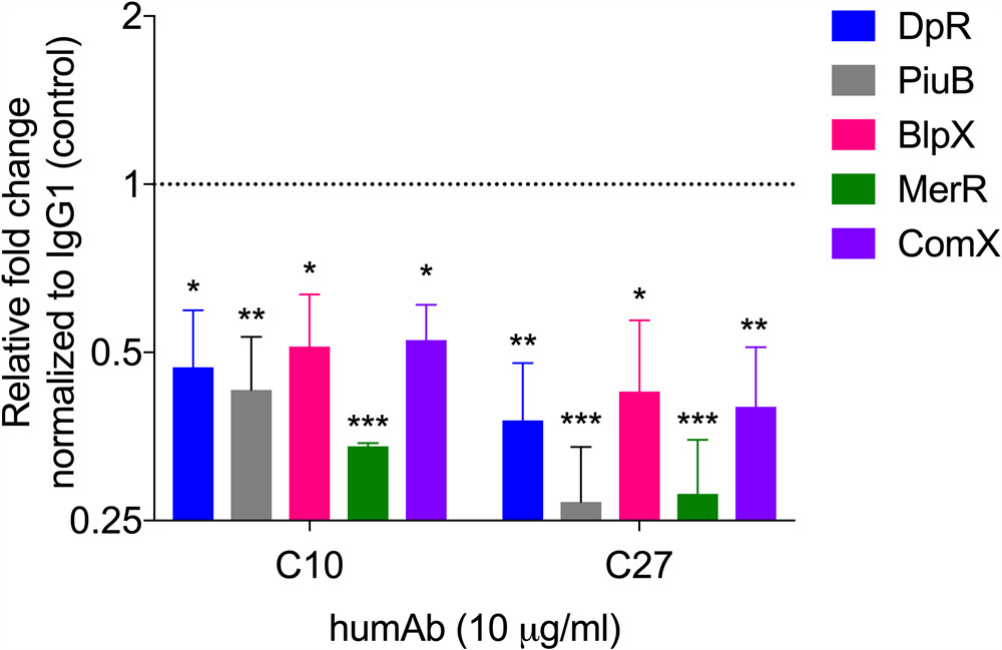
HumAbs mediate changes in the expression of bacterial genes related to oxidative stress *in vitro*. The fold change in expression of the indicated genes in C10- or C27-treated bacteria relative to the control IgG1-treated bacteria was determined by RT-qPCR at 1.5 hours post-humAb addition. The relative expression of genes was determined using the Pfaffl method (56) (fold change is relative to the IgG1 control-treated bacteria; expression, 1). Data are pooled from 3 independent experiments, with 3 samples per condition. *, *P < *0.05; **, *P < *0.01; ***, *P < *0.001, (one-way ANOVA); C10 or C27 versus IgG1.

Given that C27 did not promote agglutination or opsonophagocytosis *in vitro*, yet it reduced colonization and protected against lethal i.p. infection, we sought an alternative mechanism by which it could mediate protection. Previous work showed that certain PPS3 mAbs enhanced ST3 A66 transformation frequency and competence *in vitro*, and one mAb namely, 1E2, also altered ST3 gene expression *in vivo* (13, 16, 19). Thus, we performed reverse transcription-quantitative PCR (RT-qPCR) on reactions of ST3 A66 incubated with C10 and C27 to analyze the expression of ST3 genes that induce or respond to oxidative stress (*dpr*, *piuB*, *blpX*, *merR*, and *comX*), and of which expression was altered in 1E2-treated mice following NP colonization (16). In comparison to an IgG1 control, C10 and C27 each induced a significant decrease in *dpr* gene expression (Fig. 8). We also observed a decrease in *piuB*, *blpX*, *merR*, and *comX* expression (Fig. 8). There were no significant differences between C10 and C27 in the genes examined.

### Analysis of humAbs with V_L_ swaps.

**FIG 9:**
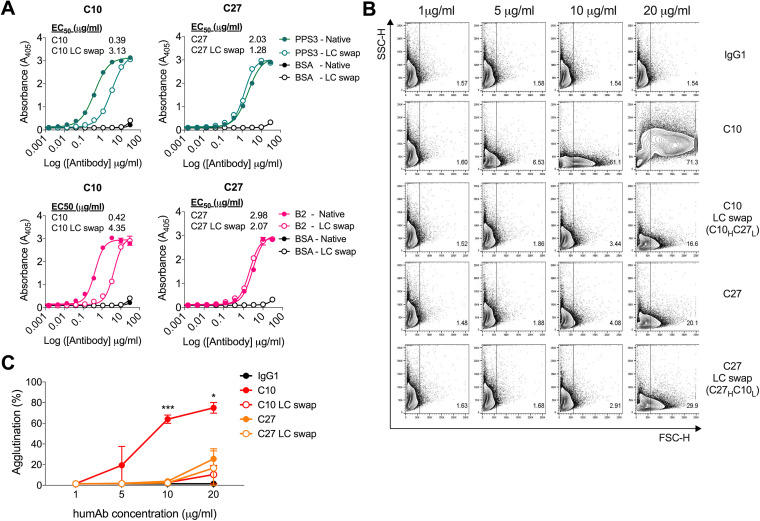
HumAb binding and agglutination of humAbs with light chain swaps. (A) HumAbs (native) with their LC swaps were generated and tested by ELISA for binding reactivity to purified PPS3 and B2. Absorbance at 405 is shown on the *y* axis for the humAb concentrations shown on the *x* axis for each humAb. The 50% effective concentration (EC_50_) is depicted on the graph. Results are representative of 3 independent experiments (*n* = 2). ST3 strain B2 was incubated with increasing concentrations of humAbs (C10, C10 LC swap [C10_H_C27_L_] or C27, C27 LC swap [C27_H_C10_L_]) or control IgG1 and analyzed by flow cytometry. (B) Representative FACS dot plots showing percent agglutination of the indicated native humAb or LC swap at various concentrations. (C) Line graph depicting percent agglutination on the *y* axis for concentrations of indicated humAbs and LC swaps on the *x* axis. Results are representative of 2 independent experiments (*n* = 2 per condition). Differences were determined by one-way ANOVA; at 10 μg/ml (C10 versus IgG1, C10 versus C10 LC swap [C10_H_C27_L_], C10 versus C27, and C10 versus C27 LC swap [C27_H_C10_L_]; ***, *P < *0.001), at 20 μg/ml (C10 versus IgG1, C10 versus C10 LC swap [C10_H_C27_L_], C10 versus C27, and C10 versus C27 LC swap [C27_H_C10_L_]; *, *P < *0.05).

**FIG 10:**
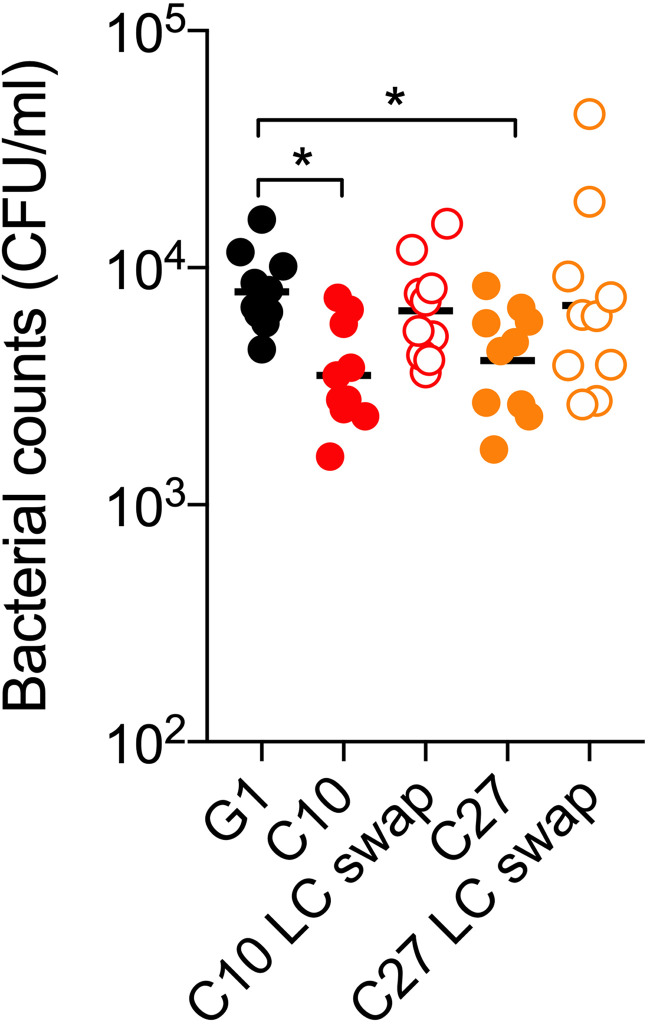
The efficacy of humAbs with light chain swaps against ST3 colonization in C57BL/6 mice. HumAbs (native) with their LC swaps or a control IgG1 were administered i.n. in C57BL/6 mice 2 h before i.n. infection with 1 × 10^6^ CFU A66. The nasal lavage CFU was enumerated 24 hours postinfection. CFUs are depicted on the *y* axis for humAbs shown on the *x* axis. Results are representative of 2 independent experiments (*n* ≥ 5 mice/group). *, *P < *0.05 (one-way ANOVA).

Given that C10 and C27 use the same V_H_ and V_L_ but C27 had lower affinity, reduced ST3 binding, and more mutations in its V_L_ region relative to the germline, we performed V_L_ swaps to evaluate the effect of V_L_ on binding and agglutination. PPS3 and B2 binding of C10 expressing the V_L_ of C27 (C10_H_C27_L_) was reduced compared to that of native C10, whereas C27 exhibited no differences in binding when expressing the C10 V_L_ (C27_H_C10_L_) (Fig. 9A). In agglutination experiments with B2, 20 μg/ml of C10 promoted strong agglutination (∼75%) compared with C10_H_C27_L_ (∼10%), but there were no differences in agglutination for C27_H_C10_L_ relative to native C27 (Fig. 9B and C). Next, we analyzed the *in vivo* efficacy of humAbs versus their LC swaps in the ST3 A66 colonization model. Compared with the IgG1 control, administration of C10 and C27 reduced NP CFUs after infection with A66 (C10, *P* = 0.0101; C27, *P* = 0.0435). However, neither the C10 nor the C27 LC swap reduced NP colonization (Fig. 10).

## DISCUSSION

Here, we report the gene use and *in vitro* functional activity of seven PPS3 humAbs generated from pneumococcal vaccine recipients. We also demonstrate the efficacy of two humAbs (C10 and C27), which use the same V_H_ and V_L_ genes (V_H_3-9*01/V_L_2-14*03), against NP colonization and lethal ST3 infection in mice. Our data show that the humAbs with the highest affinity, namely, C10, C34, and C38, mediated the most ST3 agglutination and opsonophagocytic activity. Agglutinating PPS antibodies have been reported to enhance complement activation and complement-dependent killing *in vitro* and have also been shown to be important for reducing pneumococcal colonization in mice (20, 22, 23). Notably in our study, humAb ST3 agglutination occurred at low concentrations (≤20 μg/ml), whereas other reported PPS antibodies mediated agglutination of ST14 (100 μg/ml) (22) and ST23 (250 μg/ml) (23) at much higher concentrations. It is possible that humAb agglutination could have augmented CFU reductions in the OPA, as it was observed in the absence of HL60 cells. However, we do not know if CFU reductions reflected ST3 clumping or killing (26).

Consistent with prior work demonstrating V_H_3 restriction of PPS- and other polysaccharide-binding antibodies (27–30), each humAb except C38 used a V_H_3 gene element. PPS3-specific residues important for PPS23F binding of a V_H_3-30 humAb (31) were not present in our humAbs. However, C10, C27, C38, and C11 each had Ala-Arg-Asp: ARD or Ala-Arg-Gly: ARG V_H_ CDR3 motifs, which have been described in PPS-binding (32) and polyreactive antibodies from pneumococcal vaccine recipients (33). There were no common V_L_ motifs, but the C18 V_L_ CDR3 was identical to a PPS8-binding kappa humAb that used the same V_L_ gene (V_L_ 2-30) (32). Serological cross-reactivity has not been described for PPS3 and PPS8, but they are similar structurally (34).

An in-depth analysis of C10 and C27 humAbs revealed that in contrast to C10, C27 had lower PPS3 affinity, minimal agglutinating ability, did not mediate opsonophagocytosis, and had more somatic mutations in its V_L_ relative to the germline. Nonetheless, both C10 and C27 reduced NP colonization with ST3 A66 and the clinical ST3 strain B2 (Table 2). Similarly, both humAbs prolonged survival after lethal A66 i.p. infection, suggesting that the presence of complement components and neutrophils in the blood may have enhanced the ability of the lower-affinity C27 to mediate ST3 clearance, as described for polyclonal IgG (35). However, i.n. administration of C10 but not C27 was protective against lethal i.n. challenge with B2. Even though both humAbs reduced NP colonization and inflammatory cytokines in the NP colonization model with this strain, it appears that only C10 prevented dissemination. Notably, an agglutinating mouse mAb 1E2, prevented dissemination to the lungs after NP colonization, whereas a nonagglutinating mouse mAb 7A9, did not (13). However, we do not know if the reduced efficacy of C27 in our model reflects an inability to prevent dissemination and/or distinct features of the ST3 clinical strain B2. Tissue-specific differences in virulence have been identified for other STs (36, 37), but further work is needed to dissect the roles that humAbs and ST3 strain-specific differences may play in the reduced efficacy of C27 observed in the lethal i.n. infection model.

**TABLE 2:** Summary of *in vitro* and *in vivo* functions for all PPS3 humAbs^*a*^

HumAb	*In vitro* function				*In vivo* function			
	Binding (EC_50_) of:		Agglutination	OPA	Reduction in colonization		Survival	
	PPS3	ST3			A66	B2	i.p. → i.p. challenge^*b*^ (A66) (%)	i.n. → i.n. challenge^*c*^ (B2) (%)
IgG1 control	ND	ND	−	−	N	N	8	14
C10	0.24	0.51	+	+	Y	Y	92	85
C27	2.03	11.0	−	−	Y	Y	76	29
C38	0.09	0.05	+	+	N/A	N/A	70	N/A
C11	19.30	19.20	−	−	N/A	N/A	N/A	N/A
C12	0.55	3.23	+	+	N/A	N/A	N/A	N/A
C18	1.13	2.84	−	+	N/A	N/A	N/A	N/A
C34	0.21	3.01	+	+	N/A	N/A	N/A	N/A

a ND, not detected; N/A, not applicable; Y, yes; N, no; +, strong activity; −, weak/no activity.

b i.p. → i.p. challenge, refer to Fig. 7A.

c i.n. → i.n. challenge, refer to Fig. 7B.

The main mechanism by which pneumococcal vaccine-elicited antibodies are thought to confer protection is by mediating ST-specific opsonophagocytosis, and this function has been considered a surrogate for vaccine efficacy in clinical studies (6–8). While vaccine effectiveness studies support this association for most vaccine-included STs, this is not the case for ST3 (against which current vaccines are less effective than other STs) (2). Given that our data show that C10, which was highly agglutinating and opsonophagocytic, reduced colonization and protected against lethal i.n. ST3 infection, its efficacy could stem from its agglutinating ability. On the other hand, C27, which exhibited neither agglutinating nor opsonophagocytic activity, did not protect against lethal i.n. infection. There is now ample evidence that ST-specific agglutination can reduce NP colonization in mice (13) but less evidence that opsonophagocytic antibodies reduce colonization. In fact, a PPS3 mouse mAb (7A9) that protected against lethal ST3 i.n. infection and sepsis did not reduce ST3 NP colonization in mice (11, 13). Thus, it is possible that ST-specific agglutination, which has not been examined as a correlate of pneumococcal vaccine efficacy in clinical studies, may be a better correlate of vaccine effectiveness against pneumococcal colonization and transmission than opsonophagocytosis. In support of this concept and as previously highlighted, agglutinating PPS antibodies are important in the prevention of pneumococcal colonization in mice (20, 22, 23). While this information may help explain the ability of C10 and C38 to reduce colonization in our models, it does not explain that of C27.

C27 did not exhibit agglutination or opsonophagocytosis *in vitro* but reduced colonization and prevented death from lethal i.p. infection *in vivo*. Although it may have mediated these functions *in vivo*, its lower affinity seems to make this unlikely. Given that we cannot explain its activity based on known mechanisms of PPS antibody action, we explored the possibility that C10 and C27, which both reduced colonization, may exert direct effects on ST3 as described for a mouse PPS3 mAb that altered gene expression and affected ST3 survival (16, 19). We observed downregulation of *dpr*, which is normally expressed in response to intracellular iron and needed to sequester iron to protect bacteria from oxidative damage (38–40). However, in contrast to the *in vivo* study with the mouse mAb we found that C10 and C27 also reduced expression of additional ST3 genes, including *blpX*, an immunity gene needed to avoid bacteriocin-mediated suicide and protect against other bacteriocins (41) and *piuB*, which is essential for regulating iron transport (42). Given their importance in the response to oxidative stress, it is possible that PPS3 antibody-mediated downregulation of these genes could affect ST3 survival. Along the same lines, penicillin treatment reduced expression of ST2 pneumococcal genes related to pneumococcal iron uptake (Piu) operon *piuBCDA* and competence (43). Experiments to assess the effect of these humAb-induced changes in ST3 gene expression *in vitro* on ST3 viability *in vivo* would be very informative but were beyond the scope of the current study.

The affinity differences between C10 and C27 could be related to their distinct V_H_ and V_L_ mutations. Notably, for clonally related PPS14 Fabs, the more extensively mutated V_H_ region had lower affinity (44), as did more highly mutated mouse Cryptococcus neoformans capsular polysaccharide mAbs, which also had less efficacy *in vivo* (45). Although C10 and C27 have a comparable number of mostly distinct V_H_ mutations, the C10 V_L_ (IGVL2-14*-03) is closer to the germline than C27. Given that the C10 LC swap (C10_H_C27_L_) had lower PPS3 affinity and was less agglutinating than native C10, the superior binding and efficacy of the native antibody may depend on its V_L_. We are not aware of data showing a role of light chain mutations in structure-function relationships for pneumococcal capsular polysaccharide antibodies, but studies have revealed that V_L_ gene use can dictate viral antibody-neutralizing activity (46, 47) and phosphorylcholine antibody specificity (48). Our data indicate that the C10 V_L_ may be required for its agglutinating activity, but it is not sufficient because it did not enhance the ability of C27 to agglutinate PPS3. Moreover, the ability of both humAbs to reduce A66 colonization was lost when their respective light chains were replaced, highlighting the importance of V_L_ structure and V_H_/V_L_ pairing for PPS3 agglutination and *in vivo* efficacy. Given that our data show that C10 and C27 likely bind distinct determinants, PPS3 agglutination may depend on binding to a specific PPS3 epitope (or epitopes). Understanding such interactions requires identification of PPS3 epitopes and structural requirements for antibody binding which will be important to address in the future, as recently reported for a PPS3 mouse mAb V_H_ (21).

To our knowledge, this is the first in-depth report of the binding and functional characteristics of pneumococcal vaccine-elicited PPS3 humAbs. Our findings reveal an unexpected role for the V_L_ in PPS3 binding, agglutination, and *in vivo* efficacy. Our results also confirm prior reports demonstrating the ability of PPS3 antibodies to affect ST3 gene expression *in vitro*, suggesting a possible mechanism by which nonopsonic and nonagglutinating antibody functions may translate into an ability of certain human PPS3 antibodies to reduce ST3 colonization. Although more analysis is needed to pinpoint PPS3-humAb structure-function relationships to specific determinants, our data suggest that such investigations may be useful to inform the development of therapeutic ST3 humAbs and more immunogenic ST3 vaccines, which remain urgently needed given the continued global threat of ST3 infection (1–5).

## MATERIALS AND METHODS

### Bacteria.

S. pneumoniae ST3 strain A66 (provided by David Briles; University of Alabama at Birmingham, AL) and a clinical ST3 strain, B2 (isolated in the Montefiore Medical Center [MMC] clinical microbiology laboratory under Albert Einstein College of Medicine institutional review board [IRB] protocol 2014-4035), were grown as described previously (13).

### Mice.

Six- to eight-week-old wild-type (WT) female C57BL/6 mice (NCI) were housed in the Albert Einstein College of Medicine Institute for Animal Studies (IAS). All animal studies were approved by the Institutional Animal Care and Use Committee at Albert Einstein College of Medicine (protocol number 20171212).

### PBMC blood collection.

After we obtained informed consent under Einstein/Montefiore Institutional Review Board protocol 2016-7376, peripheral blood mononuclear cells (PBMCs) were isolated by density gradient centrifugation as described (49) from whole blood of healthy volunteers at 7 days after pneumococcal vaccination (Pneumovax or Prevnar13). PBMCs were stored in liquid nitrogen prior to use.

### PPS3-PE antigen optimization.

Concentrations of fluorescently conjugated PPS3 (PPS3-PE) (Fina BioSolutions) were incubated with ST3 mouse hybridoma cells (11) with or without unlabeled PPS3 (25 μg/well). PPS3-PE-positive cells were gated by flow cytometry with cells without PPS3-PE as negative controls. The optimal concentration had a similar background fluorescence as that of control cells (see Fig. S7 in the supplemental material).

### Sorting of PPS3-binding memory B cells by flow cytometry.

PBMCs were combined from three pneumococcal vaccine recipients aged 25 to 42 (two Pneumovax and one PCV13 recipient) to increase the probability of isolating PPS3-specific memory B cells. PPS3-memory B cells were defined as CD19^+^CD27^+^IgM^−^IgG^+^PPS3^+^. PBMCs were stained with PPS3-PE and anti-human fluorescently conjugated CD19-PE-Cy7, CD27-APC, IgM-FITC, IgG-V421, CD3-V500, CD4-V500, CD8-V500, and CD14-V500 (BD). Live/dead (LD) cells were identified with the Zombie aqua fixable viability kit (Biolegend). CD3-, CD4-, CD8-, and CD14-positive cells were excluded. The gating strategy is shown in Fig. S8 in the supplemental material. Single cells were sorted on a BD FACSAria II instrument into 96-well PCR plates (MicroAmp Endura Optical 96-well clear reaction plates; Life Technologies) into lysis buffer as described (50).

### HumAb generation.

Variable heavy (V_H_) and variable light (V_L_) chain immunoglobulin genes from sorted B cells were PCR amplified, sequenced, cloned, and produced as human IgG1s in HEK-293 cells as described (50, 51). For cloning and ligation into human IgG1 expression vectors (IgG-AbVec [PBR322 based], Igκ-AbVec [PBR322 based], and Igλ-AbVec [PBR322 based] (obtained from reference 51), refined primers listed in reference 52 were used to generate DNA fragments with overlapping ends). Gibson assembly was performed to ligate DNA fragments with their corresponding digested vectors using the NEBuilder high-fidelity (HiFi) DNA assembly master mix (New England BioLabs [NEB]) according to the manufacturer’s guidelines. Sequencing of V_H_ and V_L_ regions was performed by Genewiz (New Jersey, NY). HumAbs were purified using the Gentle Ag/Ab binding and elution buffer kit (Thermo Scientific). HumAbs were concentrated using Millipore Amicon ultracentrifugal filter tubes (30K MWCO) and resuspended in 200 mM NaCl and 20 mM HEPES (pH 7.4).

### ELISA to determine binding profiles and competition assays.

PPS3 ELISAs were performed using 96-well Nunc Maxisorp plates (ThermoFisher Scientific) coated with purified PPS3 (ATCC) (10 μg/ml) in phosphate-buffered saline (PBS) overnight at 4°C as described (11, 53). Pneumococcal polysaccharide 8 (PPS8) (ATCC) (10 μg/ml) was used as a negative control. The numerical 50% effective concentration (EC_50_) was determined by GraphPad Prism software. A whole-cell ELISA (54) was used to determining binding to the clinical strain B2, which was similar to that for PPS3. Competition assays were performed as described previously (15) with some modifications. ELISA binding curves of either 1E2 or C18 on PPS3-coated plates were used to determine the concentration of antibody resulting in 50% saturation to use in the assay. This chosen concentration of either antibody (1E2 or C18) was added in equal volume to various dilutions of humAbs before addition to PPS3-coated wells. Anti-mouse IgG or anti-human IgG kappa alkaline phosphatase (AP)-conjugated secondary antibodies (Southern Biotech) were used to detect 1E2 or C18 binding, respectively, to PPS3-coated ELISA plates. The signal remained constant and was only reduced if the competing humAb bound to a similar PPS3 determinant.

### Generation of F(ab′)_2_ fragments.

F(ab′)_2_ fragments were generated using the IdeZ protease (NEB), purified using CaptureSelect LC-lambda affinity matrix (human) (ThermoFisher), and concentrated with Amicon ultracentrifugal filter tubes (30K MWCO) according to manufacturers’ instructions. Digestion and purification were confirmed by SDS-PAGE using mini-Protean TGX precast gels (4% to 20%) (Bio-Rad).

### *In vitro* agglutination of ST3 bacteria.

HumAb agglutination of ST3 was determined by flow cytometry as described (23, 55). ST3 strains A66 or B2 (1 × 10^5^ CFU) were incubated with humAbs, F(ab′)_2_ fragments, or human IgG1 (control) (Southern Biotech) for 1 h at 37°C in a 96-well plate. Cells were fixed with 1% paraformaldehyde and analyzed by flow cytometry. Bacteria were gated on forward scatter (FSC) and sideward scatter (SSC) (referring to cell size and granularity) to determine percent agglutination. Agglutination was also assessed by light microscopy. Aliquots from each sample were spotted onto 1% agarose pads and visualized with an AxioImager Z1 microscope (Zeiss).

### Immunofluorescence.

HumAbs (20 μg/ml) were mixed with 1 × 10^6^ bacteria (50 μl) in microcentrifuge tubes and incubated for 1 h at 37°C. Bacteria were washed one time with PBS by centrifugation and anti-human IgG-fluorescein isothiocyanate (FITC) was added to each sample and incubated for 1 h at 37°C. After being washed, aliquots were spotted onto 1% agarose pads and visualized with an AxioImager Z1 microscope (Zeiss) (×100 magnification).

### Opsonophagocytosis assay (OPA).

The assay was performed with differentiated HL-60 cells at an effector/target cell ratio of 400:1 as described (11, 24). HumAbs and IgG1 (control) (Southern Biotech) were diluted 3-fold from 20 μg/ml. ST3 (A66) killing (%) was determined in the presence of humAbs under the following two conditions: with HL60 cells and complement (3- to 4-week rabbit complement; Pel-Freez) or without HL60 cells (humAbs and bacteria only) by plating aliquots of samples onto blood agar plates and enumerating CFU.

### *In vitro* bacterial gene expression by reverse transcription-quantitative PCR (RT-qPCR).

To analyze the expression of selected genes during *in vitro* growth as described previously (16), in brief, bacteria were grown as described above and diluted to a starting optical density (OD) of ∼0.01. Then, 1 ml of the culture was incubated with humAbs (C10 and C27) or IgG1 control at a concentration of 10 μg/ml for 1.5 hours at 37°C. Bacterial RNA was extracted using the TRIzol Max bacterial RNA isolation kit (Life Technologies) using the manufacturer’s protocol. RNA was then DNase treated using the Turbo DNA-free kit (Invitrogen), and cDNA was synthesized from 200-ng RNA using the iScript cDNA synthesis kit (Bio-Rad). qPCR was performed using Power SYBR green master mix (Life Technologies) with 10-ng cDNA and 10-μm primers as outlined in Table S1 in the supplemental material, as per the manufacturer’s instructions. Amplification was performed on a StepOne Plus real-time PCR system (Life Technologies) using the following conditions: 95°C for 10 min followed by 40 cycles of 95°C for 15 seconds and 60°C for 1 min. The relative expression of genes in humAb-treated bacteria was calculated using the threshold cycle (2ΔΔ*C_T_*) method as described previously (56) using the 16S rRNA gene as an internal control and control IgG1-treated bacteria as the reference.

### Mouse infection experiments.

For the colonization model, mice were anesthetized with isofluorane and injected intranasally (i.n.) with 25 μg of humAbs or anti-human IgG1 (Bxcell) (isotype control) diluted in PBS as described (13). Two hours after humAb administration, mice were infected i.n. with either 5 × 10^5^ to 1 × 10^6^ CFU of A66 or 1 × 10^7^ CFU of B2 in 10 μl. CFUs were enumerated in the nasal lavage (NL) and lungs at the times specified (24 h or 4 days) after infection as described (13). NL cytokines were determined after concentration using the Legendplex mouse inflammation panel (13-plex) (Biolegend) as per the manufacturer’s protocol.

For the lethal infection model, mice were injected either i.p. or i.n. with 25-μg humAb or anti-human IgG1 in PBS as described above. Two hours after humAb administration, mice were infected i.p. with 5 × 10^5^ CFU A66 (100 μl) or i.n. with 5 × 10^7^ CFU B2 in 10 μl and monitored for survival. Dose-response experiments were performed to determine lethal doses for use in the study.

### Statistical analysis.

Data were analyzed using a Fisher’s exact test (survival) or a one-way analysis of variance (ANOVA) for other analyses as indicated in the figure legends using GraphPad Prism. *P* values of ≤0.05 were considered significant.

### Data availability.

GenBank accession numbers were as follows: C10V_H_, MZ054262; C11V_H_, MZ054263; C12V_H_, MZ054264; C18V_H_, MZ054265; C27V_H_, MZ054266; C34V_H_, MZ054267; C38V_H_, MZ054268; C10V_L_, MZ054269; C11V_L_, MZ054270; C12V_L_, MZ054271; C18V_L_, MZ054272;, C27V_L_, MZ054273;, C34V_L_, MZ054274; and C38V_L_, MZ054275.
