# Supplementary material for: Isolation and Characterization of Human Monoclonal Antibodies to Pneumococcal Capsular Polysaccharide 3
Source: Microbiol Spectr. 2021 Nov 10;9(3):e01446-21. doi: 10.1128/Spectrum.01446-21 (PMC8579928; doi:10.1128/Spectrum.01446-21)
Supplement: SUPPLEMENTAL FILE 1 — Supplemental material. Download SPECTRUM01446-21_Supp_1_seq12.pdf, PDF file, 1.9 MB [file spectrum01446-21_supp_1_seq12.pdf]

SUPPLEMENTARY MATERIAL

TABLES:

Table S1. Primers used for RT-qPCR

Primer Name	Sequence (5’-3’)
**16s-F**	ATAGCCGACCTGAGAGGGTGA
**16s-R**	TACAAGCCAGAGAGCCGCTT
**blpX-F**	GCTAATAAAGGGGTGGCGG
**blpX-R**	CCTCGCATCCAAACCAAGAT
**comX-F**	GGCATGCTCTGCTTACATGAA
**comX-R**	GGGTTCTTTATCGTATCTACGCTTC
**dpr-F**	CTCACGTTGCTTTGCACCAA
**dpr-R**	GAGAATGGGCTTCCACCGAG
**piuB-F**	GCAGACCTGGCTCCTCTTTC
**piuB-R**	ACTCGGTGCAGCAAACTGAT
**merR-F**	ACCAAGCCTCTCCTCTCTCG
**merR-R**	TCAGGATATCGAAGCGCTGG

FIGURES:

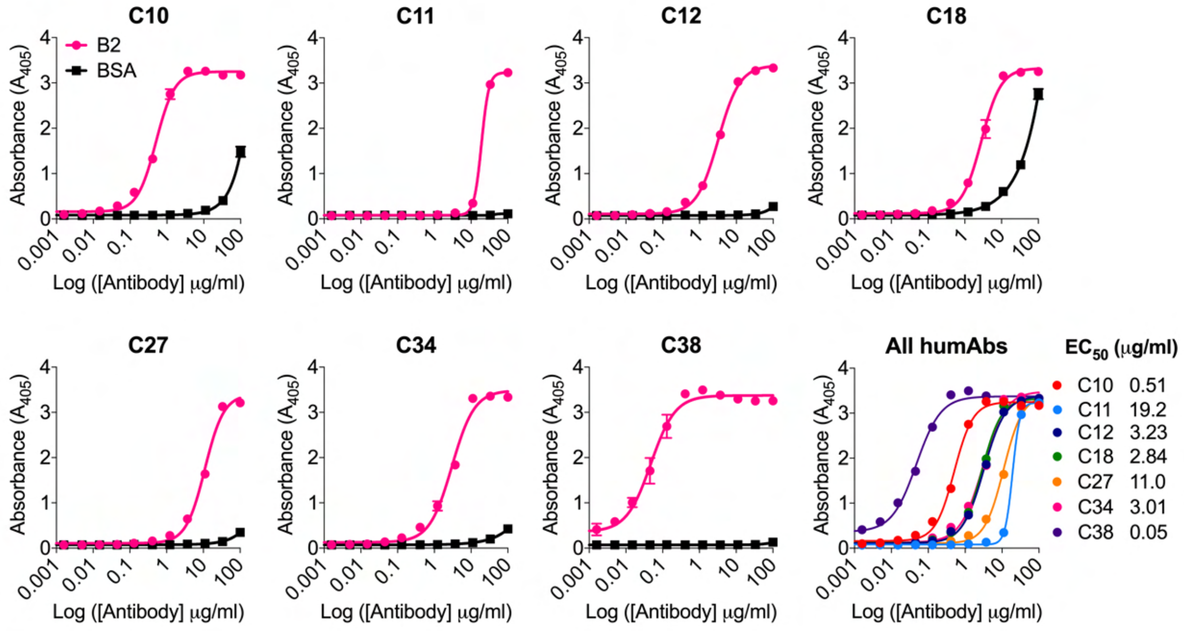

<u>Figure S1:</u> HumAb binding to ST3 clinical strain B2 by ELISA.

Binding reflected by absorbance at 405 is shown on the Y axis for the humAb concentrations shown on the X axis for each humAb. Results are representative of 3 independent experiments (n = 2). The numerical half-maximal binding titer (EC_{50}) for each humAb is indicated to the right of the panel depicting binding curves of all humAbs.

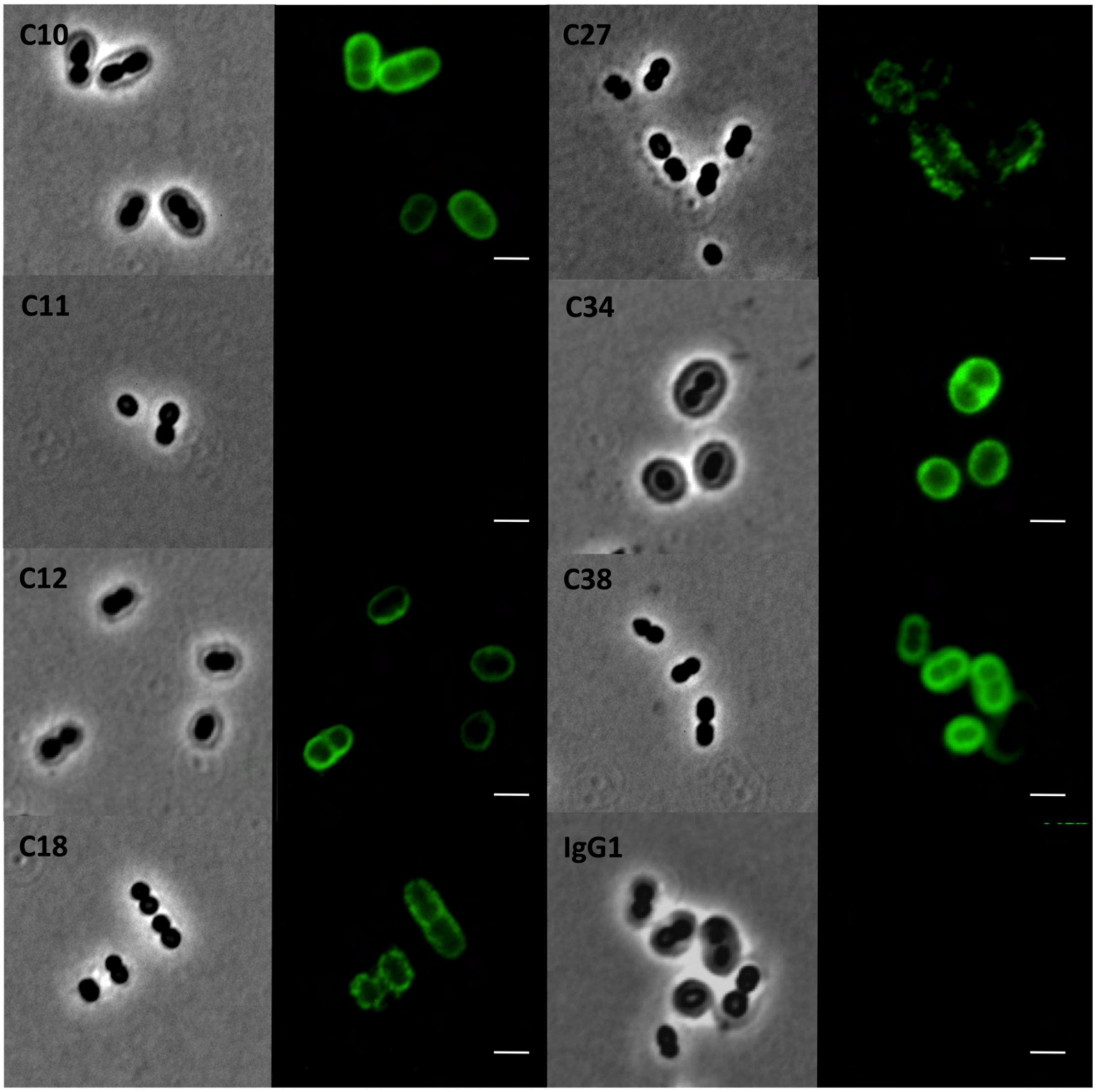

<u>Figure S2:</u> Binding of humAbs to the clinical strain B2 by immunofluorescence.
ST3 clinical strain B2 was incubated with humAbs (20 μg/ml) and antibody binding was detected using IgG conjugated to FITC. Fluorescent images were analysed at 100X magnification and representative of 2 independent experiments (n = 2). Non-specific IgG1 was used as a control. White bar represents 2μM.

V_H Regions

```
                       FR1-IMGT                   CDR1-IMGT    FR2-IMGT          CDR2-IMGT  FR3-IMGT
                       (1-26)                     (27-38)      (39-55)           (56-65)    (66-104)

                       1        10        20         30        40        50          60         70        80        90        100
                       .........|.........|......  ...|........ .|.........|..... ....|..... ....|.........|.........|.........|....
GERMLINE: IGHV3-9*01   EVQLVESGG.GLVQPGRSLRLSCAAS  GFTF....DDYA MHWVRQAPGKGLEWVSG ISWN..SGSI GYADSVK.GRFTISRDNAKNSLYLQMNSLRAEDTALYYC
C10 VH                 ---------.------T---------  ----....---- ------V---------D M--K..--I- N------.----------------------V----V---

                       1        10        20         30        40        50          60         70        80        90        100
                       .........|.........|......  ...|........ .|.........|..... ....|..... ....|.........|.........|.........|....
GERMLINE: IGHV3-30*03  QVQLVESGG.GVVQPGRSLRLSCAAS  GFTF....SSYG MHWVRQAPGKGLEWVAV ISYD..GSNK YYADSVK.GRFTISRDNSKNTLYLQMNSLRAEDTAVYYC
C11 VH                 ---------.----------------  ----....-T-V I--A-----R------L --S-..---- -------.----------E---S-------S--------

                       1        10        20         30        40        50          60         70        80        90        100
                       .........|.........|......  ...|........ .|.........|..... ....|..... ....|.........|.........|.........|....
GERMLINE: IGHV3-23*04  EVQLVESGG.GLVQPGGSLRLSCAAS  GFTF....SSYA MSWVRQAPGKGLEWVSA ISGS..GGST YYADSVK.GRFTISRDNSKNTLYLQMNSLRAEDTAVYYC
C12 VH                 ---------.--------------V-  ----....---- ---------R------T ----..-AD- -------.----------R--------N-------F---

                       1        10        20         30        40        50          60         70        80        90        100
                       .........|.........|......  ...|........ .|.........|..... ....|..... ....|.........|.........|.........|....
GERMLINE: IGHV3-7*01   EVQLVESGG.GLVQPGGSLRLSCAAS  GFTF....SSYW MSWVRQAPGKGLEWVAN IKQD..GSEK YYVDSVK.GRFTISRDNAKNSLYLQMNSLRAEDTAVYYC
C18 VH                 ---------.----------------  --S-....--S- -C--------RF----- --N-..-TD- -------.----V----------------------T---

                       1        10        20         30        40        50          60         70        80        90        100
                       .........|.........|......  ...|........ .|.........|..... ....|..... ....|.........|.........|.........|....
GERMLINE: IGHV3-9*01   EVQLVESGG.GLVQPGRSLRLSCAAS  GFTF....DDYA MHWVRQAPGKGLEWVSG ISWN..SGSI GYADSVK.GRFTISRDNAKNSLYLQMNSLRAEDTALYYC
C27 VH                 ---------.A-------------V-  ---V....E--- ----------------- S--K..--M- R------.-------------------------------

                       1        10        20         30        40        50          60         70        80        90        100
                       .........|.........|......  ...|........ .|.........|..... ....|..... ....|.........|.........|.........|....
GERMLINE: IGHV3-72*01  EVQLVESGG.GLVQPGGSLRLSCAAS  GFTF....SDHY MDWVRQAPGKGLEWVGR TRNKANSYTT EYAASVK.GRFTISRDDSKNSLYLQMNSLKTEDTAVYYC
C34 VH                 -----Q---.------E---------  ----....---F --------------I-- I----Y---- Y------.----V--------------------------

                       1        10        20         30        40        50          60         70        80        90        100
                       .........|.........|......  ...|........ .|.........|..... ....|..... ....|.........|.........|.........|....
GERMLINE: IGHV1-18*01  QVQLVQSGA.EVKKPGASVKVSCKAS  GYTF....TSYG ISWVRQAPGQGLEWMGW ISAY..NGNT NYAQKLQ.GRVTMTTDTSTSTAYMELRSLRSDDTAVYYC
C38 VH                 ---------.---E------------  ----....NN-N FT--------------- --S-..---- -----FR.--------I--T-------------------
```

<u>Figure S3</u>: Heavy chain variable region (V_H) sequences of PPS3-specific humAbs.
HumAb V_H sequences aligned with their germline counterparts based on IMGT/V-QUEST (sequence alignment software). Amino acid changes resulting from somatic mutations are indicated within the sequence alignment.

V$_L$ Regions

```
                          FR1-IMGT                 CDR1-IMGT      FR2-IMGT         CDR2-IMGT                 FR3-IMGT
                           (1-26)                   (27-38)        (39-55)          (56-65)                  (66-104)

                          1        10         20    30            40       50         60          70        80        90        100
                          .........|.........|......  ...|........  .|.........|.....  ....|.....  ....|.........|.........|.........|....
GERMLINE: IGLV2-14*03     QSALTQPAS.VSGSPGQSITISCTGT SSDVG...GYNY VSWYQQHPGKAPKLMIY DV.......S NRPSGVS.NRFSGSK..SGNTASLTISGLQAEDEADYYC
C10 VL                    --V------.---------------- N----...---- ----------V-----F --.......T -------.-------..----------------------

                          1        10         20    30            40       50         60          70        80        90        100
                          .........|.........|......  ...|........  .|.........|.....  ....|.....  ....|.........|.........|.........|....
GERMLINE: IGKV2-28*01     DIVMTQSPLSLPVTPGEPASISCRSS QSLLHS.NGYNY LDWYLQKPGQSPQLLIY LG.......S NRASGVP.DRFSGSG..SGTDFTLKISRVEAEDVGVYYC
C11 VL                    -----------Y-----------M-- -----G.----- -A----------R---- --.......- -------.-------..----------------------

                          1        10         20    30            40       50         60          70        80        90        100
                          .........|.........|......  ...|........  .|.........|.....  ....|.....  ....|.........|.........|.........|....
GERMLINE: IGLV4-69*01     QLVLTQSPS.ASASLGASVKLTCTLS SGHS.....SYA IAWHQQQPEKGPRYLMK LNSD...GSH SKGDGIP.DRFSGSS..SGAERYLTISSLQSEDEADYYC
C12 VL                    -S-------.---------------- --D-.....--T ---Y------------- ----...--- -------.-------..--S-------------------

                          1        10         20    30            40       50         60          70        80        90        100
                          .........|.........|......  ...|........  .|.........|.....  ....|.....  ....|.........|.........|.........|....
GERMLINE: IGKV2-30*01     DVVMTQSPLSLPVTLGQPASISCRSS QSLVYS.DGNTY LNWFQQRPGQSPRRLIY KV.......S NRDSGVP.DRFSGSG..SGTDFTLKISRVEAEDVGVYYC
C18 VL                    -IQ----------------------- ----F-.----- ----------------S --.......- --E----.-K-----..---------T--------L---

                          1        10         20    30            40       50         60          70        80        90        100
                          .........|.........|......  ...|........  .|.........|.....  ....|.....  ....|.........|.........|.........|....
GERMLINE: IGLV2-14*03     QSALTQPAS.VSGSPGQSITISCTGT SSDVG...GYNY VSWYQQHPGKAPKLMIY DV.......S NRPSGVS.NRFSGSK..SGNTASLTISGLQAEDEADYYC
C27 VL                    --V------.---------------- -R---...T--- ---------TV---L-F --.......T -------.D------..--D---------------E---

                          1        10         20    30            40       50         60          70        80        90        100
                          .........|.........|......  ...|........  .|.........|.....  ....|.....  ....|.........|.........|.........|....
GERMLINE: IGLV2-14*01     QSALTQPAS.VSGSPGQSITISCTGT SSDVG...GYNY VSWYQQHPGKAPKLMIY EV.......S NRPSGVS.NRFSGSK..SGNTASLTISGLQAEDEADYYC
C34 VL                    --V------.---------------- -----...N-D- ----------------N Q-.......- -------.-------..----------------------

                          1        10         20    30            40       50         60          70        80        90        100
                          .........|.........|......  ...|........  .|.........|.....  ....|.....  ....|.........|.........|.........|....
GERMLINE: IGLV1-51*02     QSVLTQPPS.VSAAPGQKVTISCSGS SSNI....GNNY VSWYQQLPGTAPKLLIY EN.......N KRPSGIP.DRFSGSK..SGTSATLGITGLQTGDEADYYC
C38 VL                    ---------.---------------T T---....E--- ----------------- G-.......- -------.-------..----------------------
```

Figure S4: Light chain variable region (V$_L$) sequences of PPS3-specific humAbs.
HumAb V$_L$ sequences aligned with their germline counterparts based on IMGT/V-QUEST (sequence alignment software). Amino acid changes resulting from somatic mutations are indicated within the sequence alignment.

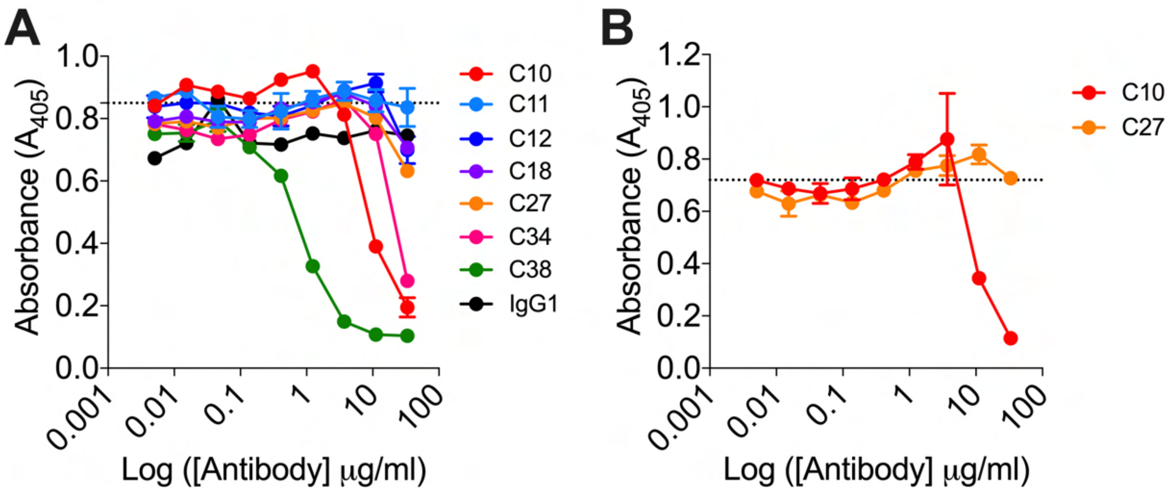

Figure S5: Competition ELISA with humAbs

Competition ELISAs were performed with humAbs. Varying concentrations of specified humAbs were added to a fixed concentration of A) 1E2 or B) C18. Binding of either 1E2 or C18 is reflected by absorbance at 405 shown on the Y axis when incubated with the specified dilution of humAb shown on the X axis. The dotted line represents average signal obtained with fixed concentration of 1E2 or C18 alone. Competition is depicted by signal reduction. Results are representative of 2 independent experiments (n = 2).

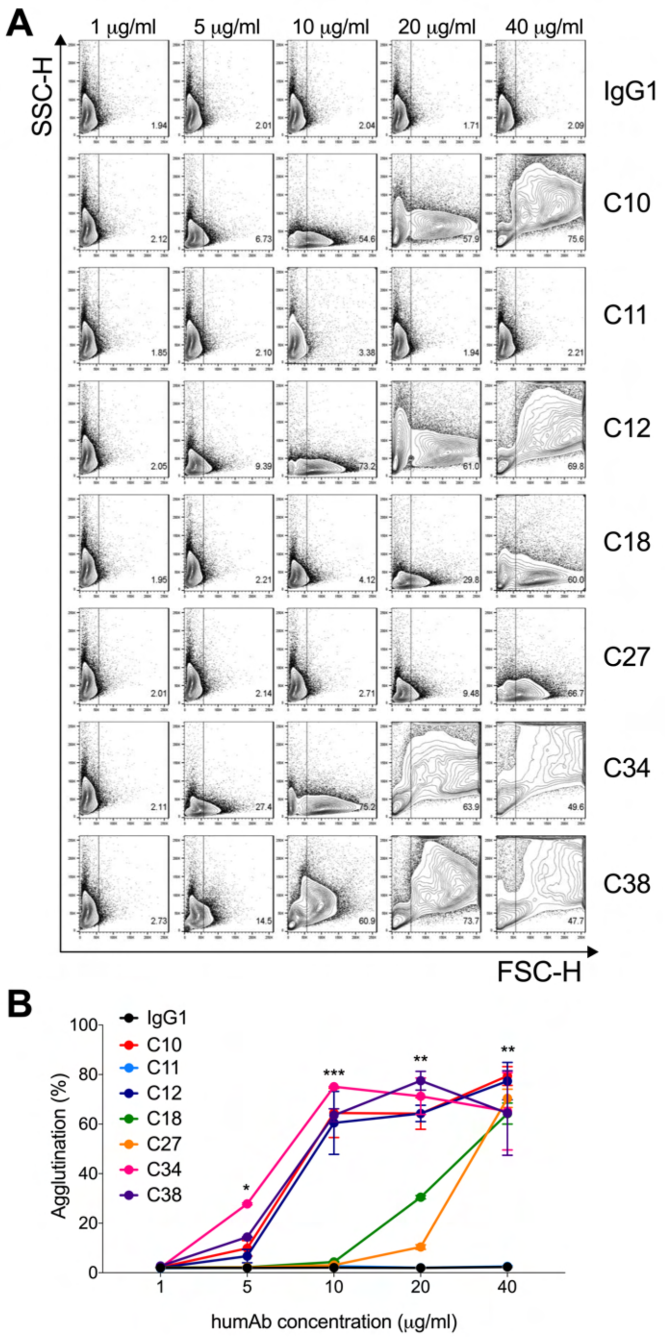

Figure S6: ***In vitro*** **agglutination of a clinical ST3 strain.**
HumAbs were tested for their ability to agglutinate a ST3 clinical strain (B2) by flow cytometry. A) Representative FACS dot plots showing the percentage agglutination of all humAbs and control human IgG1 at various concentrations by flow cytometry B) Percentage of agglutination is shown on the Y axis for different humAb concentrations indicated on the X axis. Graph represents data from 2 independent experiments (n = 2 per condition). By one way ANOVA: At 5 µg/ml; (C10, C34 & C38 vs IgG1 *P<0.05), at 10 µg/ml; (C10, C12, C34 & C38 vs IgG1 ***P<0.001); at 20 µg/ml (C10, C12, C18, C34 & C38 vs IgG1 **P<0.01), at 40 µg/ml (C10, C12, C18, C27 & C38 vs IgG1 **P<0.01).

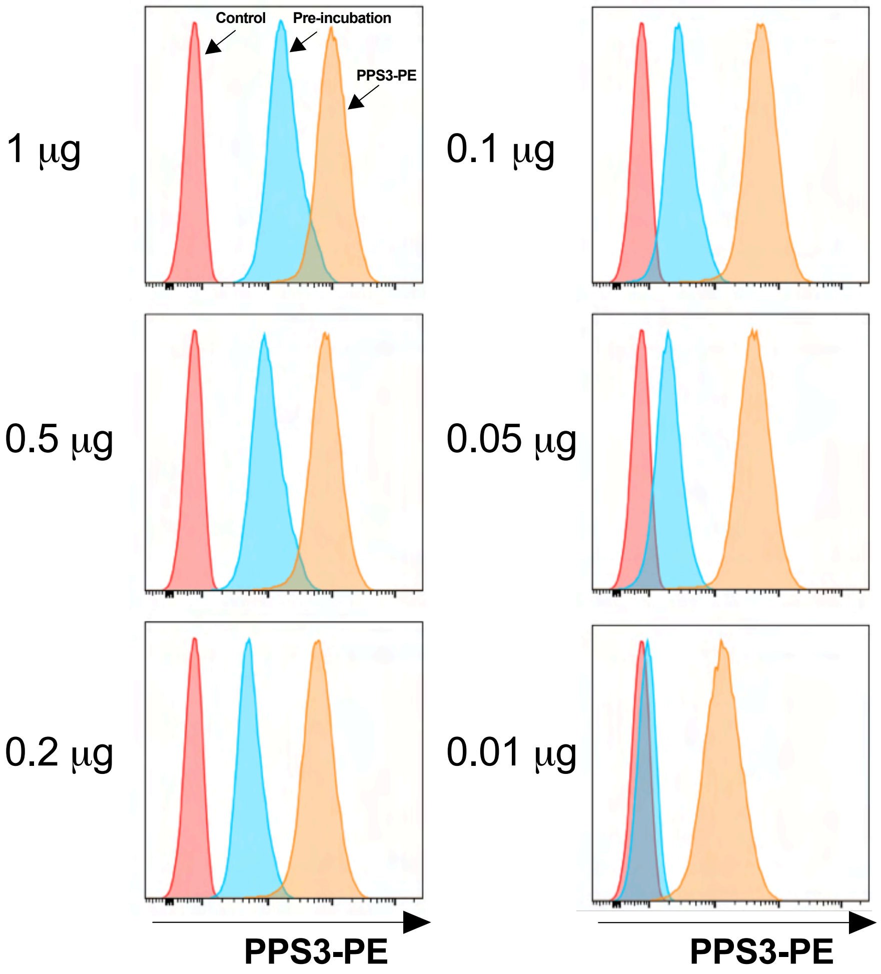

<u>Figure S7:</u> **PPS3-PE optimization with ST3 hybridoma cells.**
ST3 hybridoma cells were optimized with different concentrations of PPS3-PE with and without the presence of unlabelled PPS3 (25 µg/well). PPS3-PE positive signal was determined by flow cytometry. Histograms represent the following groups; Control (cells with no PPS3-PE) (Red), Pre-incubation (cells pre-incubated with unlabelled PPS3 prior to addition of PPS3-PE) (blue), PPS3-PE (cells incubated only with PPS3-PE) (Orange). Results are representative of 2 independent experiments (n = 2).

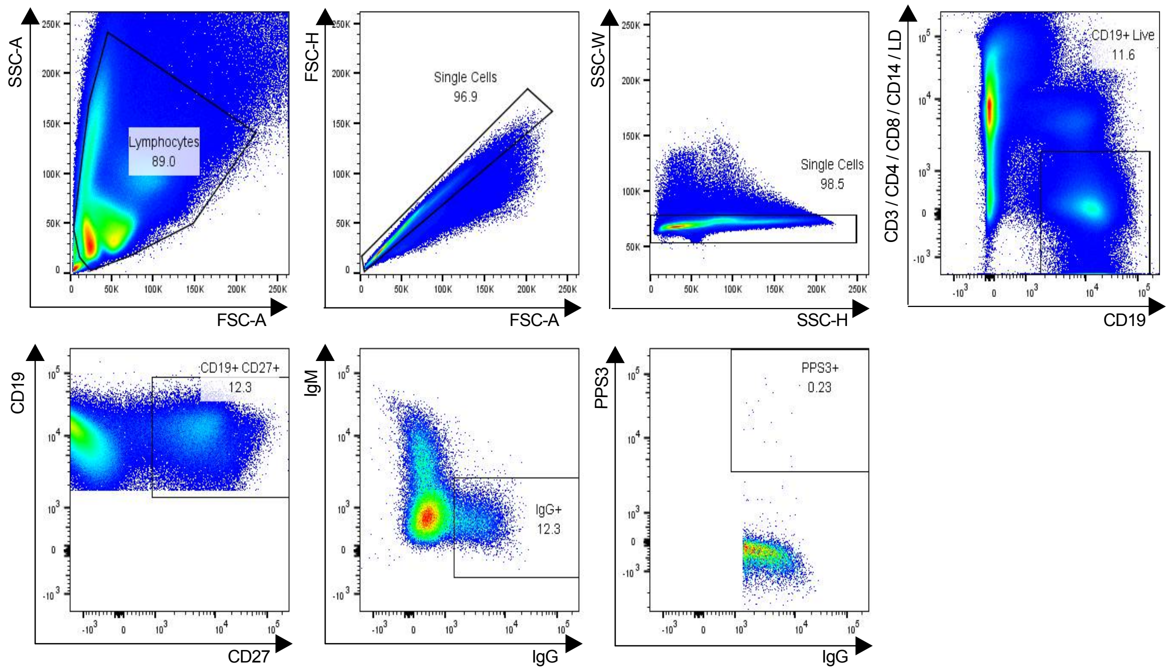

<u>Figure S8:</u> Representative gating strategy to sort for PPS3+ Memory B cells.
PBMCs were collected from patients 7 days following vaccination with pneumococcal vaccines and stained to sort for $CD19^{+}CD27^{+}IgM^{-}IgG^{+}PPS3^{+}$ cells (See Methods for details).
